# Supplementary material for: Differential dynamics of microbial community networks help identify microorganisms interacting with residue-borne pathogens: the case of Zymoseptoria tritici in wheat
Source: Microbiome. 2019 Aug 30;7:125. doi: 10.1186/s40168-019-0736-0 (PMC6717385; doi:10.1186/s40168-019-0736-0)
Supplement: Supplementary file 1 — Table S1. Number of ASVs detected for each analysis performed on the dataset and properties of residue microbial ecological networks. (DOCX 27 kb) [file 40168_2019_736_MOESM1_ESM.docx]

**Additional file 1: Table S1** - Number of ASVs detected for each analysis performed on the dataset and properties of residue microbial ecological networks

|  |  |  | All taxa | | Number of taxa promoted in inoculated condition | | | Number of taxa promoted in non-inoculated condition | | | Number of taxa promoted in contact with the soil (SC) | | | Number of taxa promoted without contact with the soil (AG) | | | Network analysis | | | | | |
| --- | --- | --- | --- | --- | --- | --- | --- | --- | --- | --- | --- | --- | --- | --- | --- | --- | --- | --- | --- | --- | --- | --- |
| Year | Sampling date | Soil condition | F | B | F | B | Total | F | B | Total | F | B | Total | F | B | Total | F | B | Total | Interacting node | Isolated node | *Z. tritici* interaction |
| 2016-2017 | Jul. | - | 36 | / | 8 | / | 8 | 6 | / | 6 | - | - | - | - | - | - | 20 | / | 20 | 12 | 8 | 2 |
| 2016-2017 | Oct. | AG | 61 | 182 | 8 | 2 | 10 | 4 | 1 | 5 | 13 | 31 | 44 | 9 | 18 | 27 | 32 | 73 | 105 | 79 | 26 | 4 |
| 2016-2017 | Oct. | SC | 106 | 167 | 1 | 2 | 3 | 3 | 6 | 9 |  |  |  |  |  |  | 52 | 90 | 142 | 133 | 9 | nd |
| 2016-2017 | Dec. | AG | 101 | 260 | 7 | 4 | 11 | 5 | 4 | 9 | 13 | 36 | 49 | 6 | 16 | 22 | 39 | 100 | 139 | 121 | 18 | nd |
| 2016-2017 | Dec. | SC | 164 | 227 | 2 | 3 | 5 | 0 | 4 | 4 |  |  |  |  |  |  | 51 | 105 | 156 | 150 | 6 | nd |
| 2016-2017 | Feb. | AG | 138 | 285 | 2 | 2 | 4 | 1 | 1 | 2 | 13 | 37 | 50 | 14 | 20 | 34 | 48 | 107 | 155 | 145 | 10 | nd |
| 2016-2017 | Feb. | SC | 179 | 238 | 4 | 2 | 6 | 1 | 1 | 2 |  |  |  |  |  |  | 55 | 110 | 165 | 158 | 7 | nd |
| 2017-2018 | Jul. | - | 36 | 286 | 2 | 7 | 9 | 6 | 3 | 9 | - | - | - | - | - | - | 16 | 60 | 76 | 60 | 16 | 6 |
| 2017-2018 | Oct. | AG | 87 | 197 | 5 | 6 | 11 | 7 | 3 | 10 | 8 | 36 | 44 | 5 | 20 | 25 | 39 | 93 | 132 | 119 | 13 | 7 |
| 2017-2018 | Oct. | SC | 117 | 216 | 4 | 6 | 10 | 5 | 2 | 7 |  |  |  |  |  |  | 31 | 109 | 140 | 126 | 14 | 2 |
| 2017-2018 | Dec. | AG | 104 | 252 | 5 | 13 | 18 | 4 | 9 | 13 | 7 | 43 | 50 | 6 | 22 | 28 | 42 | 110 | 152 | 142 | 10 | nd |
| 2017-2018 | Dec. | SC | 158 | 230 | 1 | 4 | 5 | 5 | 2 | 7 |  |  |  |  |  |  | 35 | 116 | 151 | 140 | 11 | nd |
| 2017-2018 | Feb. | AG | 135 | 340 | 6 | 8 | 14 | 2 | 6 | 8 | 8 | 33 | 41 | 10 | 21 | 31 | 44 | 119 | 163 | 152 | 11 | nd |
| 2017-2018 | Feb. | SC | 193 | 253 | 0 | 4 | 4 | 3 | 4 | 7 |  |  |  |  |  |  | 46 | 114 | 160 | 147 | 13 | nd |
