# Supplementary material for: Differential dynamics of microbial community networks help identify microorganisms interacting with residue-borne pathogens: the case of Zymoseptoria tritici in wheat
Source: Microbiome. 2019 Aug 30;7:125. doi: 10.1186/s40168-019-0736-0 (PMC6717385; doi:10.1186/s40168-019-0736-0)
Supplement: Supplementary file 2 — Table S2. Analysis of the proportion of intra-kingdom interactions (between two fungal ASVs and between two bacterial ASVs) and inter-kingdom interactions (between a fungal ASV and a bacterial ASV) in the ecological networks. The statistical significance of the under- or over-representation of inter-kingdom interactions (when F-B residuals < 1 or > 1, respectively) was established by a χ2 test of independence performed on the contingency table (χ2 < 0.001). (DOCX 33 kb) [file 40168_2019_736_MOESM2_ESM.docx]

**Additional file 2: Table S2** - Analysis of the proportion of intra-kingdom interactions (between two fungal ASVs and between two bacterial ASVs) and inter-kingdom interactions (between a fungal ASV and a bacterial ASV) in the ecological networks. The statistical significance of the under- or over-representation of inter-kingdom interactions (when F-B residuals < 1 or > 1, respectively) was established by a χ^2^ test of independence performed on the contingency table (χ^2^ < 0.001).

| Networks ^1^ | Number of species | | Number of interactions | | | Theoretical maximum number of interactions | | | Residuals | | |
| --- | --- | --- | --- | --- | --- | --- | --- | --- | --- | --- | --- |
|  | F ^2^ | B ^3^ | F-F | B-B | F-B | F-F ^4^ | B-B ^5^ | F-B ^6^ | F-F | B-B | F-B |
| Oct. 2016-2017, above ground | 32 | 73 | 17 | 57 | 17 | 496 | 2628 | 2336 | 1.856 | 0.545 | -1.939 |
| Oct. 2016-2017, contact with soil | 52 | 90 | 32 | 121 | 69 | 1326 | 4005 | 4680 | 1.060 | -0.736 | 0.358 |
| Dec. 2016-2017, above ground | 39 | 100 | 19 | 86 | 42 | 741 | 4950 | 3900 | 0.340 | 0.036 | -0.266 |
| Dec. 2016-2017, contact with soil | 51 | 105 | 25 | 145 | 74 | 1275 | 5460 | 5355 | -0.772 | 0.236 | 0.160 |
| Feb. 2016-2017, above ground | 48 | 107 | 30 | 110 | 65 | 1128 | 5671 | 5136 | 1.109 | -0.866 | 0.508 |
| Feb. 2016-2017, contact with soil | 55 | 110 | 32 | 136 | 90 | 1485 | 5995 | 6050 | 0.208 | -1.170 | 1.505 |
| July 2017-2018 | 16 | 60 | 8 | 45 | 9 | 120 | 1770 | 960 | 0.216 | 1.476 | -2.201 |
| Oct. 2017-2018, above ground | 39 | 93 | 28 | 93 | 62 | 741 | 4278 | 3627 | 1.309 | -1.321 | 1.019 |
| Oct. 2017-2018, contact with soil | 31 | 109 | 17 | 140 | 43 | 465 | 5886 | 3379 | -1.413 | 2.172 | -2.144 |
| Dec. 2017-2018, above ground | 42 | 110 | 22 | 119 | 68 | 861 | 5995 | 4620 | -0.598 | -0.253 | 0.733 |
| Dec. 2017-2018, contact with soil | 35 | 116 | 19 | 149 | 50 | 595 | 6670 | 4060 | -1.384 | 1.949 | -1.849 |
| Feb. 2017-2018, above ground | 44 | 119 | 27 | 136 | 72 | 946 | 7021 | 5236 | -0.207 | -0.081 | 0.244 |
| Feb. 2017-2018, contact with soil | 46 | 114 | 26 | 135 | 91 | 1035 | 6441 | 5244 | -0.752 | -0.978 | 1.845 |

^1^ according to sampling date, cropping season and contact with soil

^2^ fungal species

^3^ bacterial species

^4^ estimated by $C_{2}^{n_{F}}=\frac{n_{F}!}{2 \times\left( n_{F}-2 \right)!}$

^5^ estimated by $C_{2}^{n_{B}}$

^6^ estimated by $n_{F}\times n_{B}$
