# Supplementary material for: Differential dynamics of microbial community networks help identify microorganisms interacting with residue-borne pathogens: the case of Zymoseptoria tritici in wheat
Source: Microbiome. 2019 Aug 30;7:125. doi: 10.1186/s40168-019-0736-0 (PMC6717385; doi:10.1186/s40168-019-0736-0)
Supplement: Supplementary file 3 — Table S3. Sequence filtering for each run. (DOCX 24 kb) [file 40168_2019_736_MOESM3_ESM.docx]

**Additional file 3: Table S3** - Sequence filtering for each run

| Run | Primers | Sequence number (paired end) | Sequence quality trimming (F/R) | Selection by sequence length | Quality sequence number after DADA2 analysis |
| --- | --- | --- | --- | --- | --- |
| #1 | ITS1F / ITS2 | 10 536 086 (×2) | 220 / 210 | - | 7 164 826 |
| #2 | 515f / 806r | 8 368 872 (×2) | 230 / 200 | 253 bp | 5 562 335 |
| #3 | ITS1F / ITS2 | 10 216 508 (×2) | 220 / 190 | - | 6 965 664 |
| #4 | 515f / 806r | 9 975 344 (×2) | 220 / 170 | 253 bp | 5 734 825 |
