# Supplementary figures and images for: Differential dynamics of microbial community networks help identify microorganisms interacting with residue-borne pathogens: the case of Zymoseptoria tritici in wheat
Source: Microbiome. 2019 Aug 30;7:125. doi: 10.1186/s40168-019-0736-0 (PMC6717385; doi:10.1186/s40168-019-0736-0)

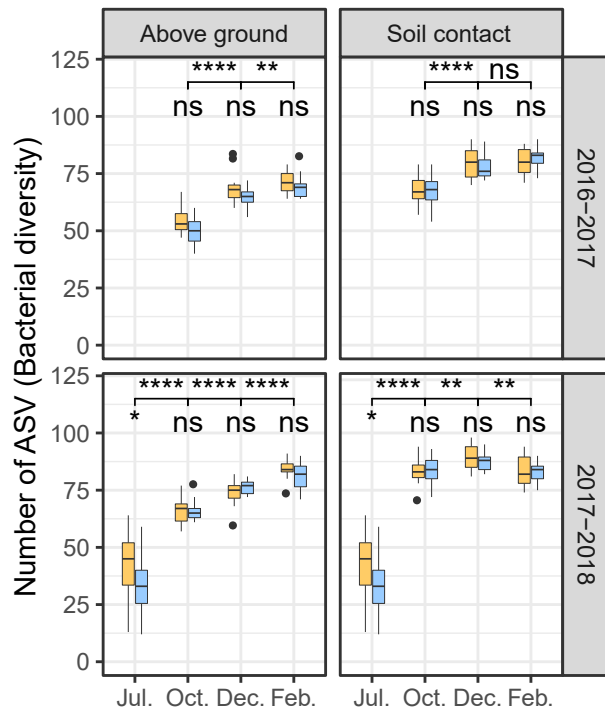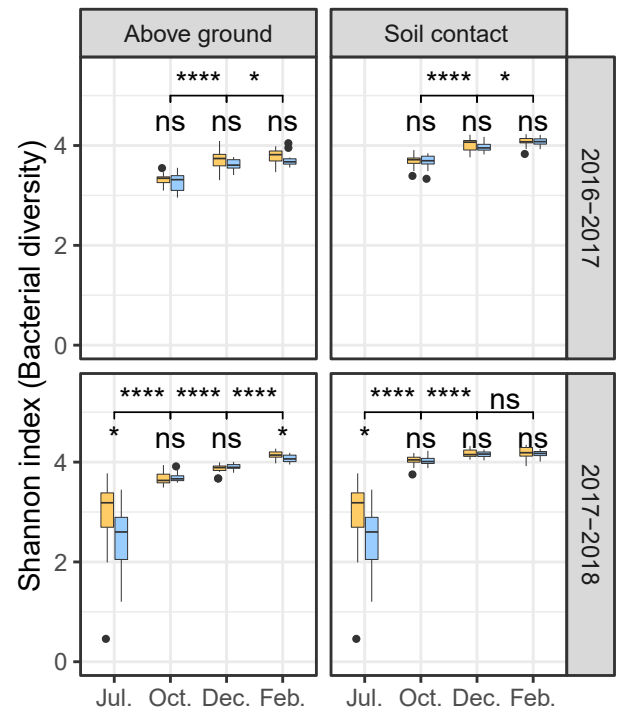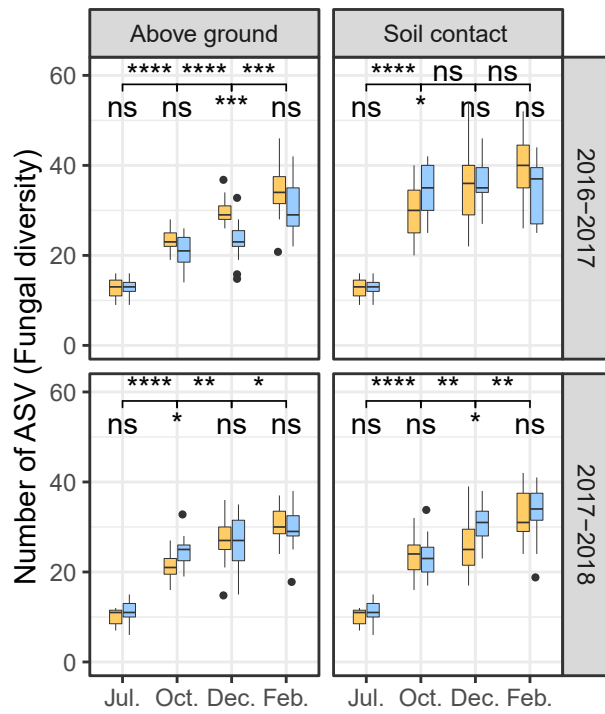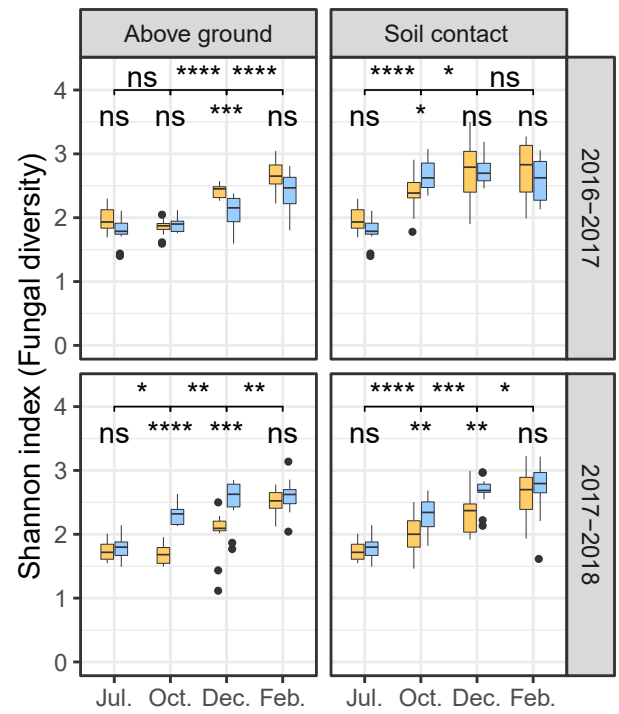

Inoculated

Non inoculated

Supplement: Supplementary file 4 — Figure S1. Alpha diversity of microbial communities associated with residues. Observed richness (number of ASVs) and diversity (Shannon index), in four sets of experimental conditions (cropping season, contact with soil, seasonality, Zymoseptoria tritici inoculation). Each box represents the distribution of the number of ASVs and Shannon index for 15 sampling points per treatment. Wilcoxon tests were performed for inoculation condition (inoculated, non-inoculated) and sampling date (July, October, December, February). Wilcoxon tests were performed for inoculation condition, and between sampling dates (NS: not significant; * p-value < 0.05; ** p-value < 0.01; *** p-value < 0.001). (PDF 120 kb) [file 40168_2019_736_MOESM4_ESM.pdf]

A

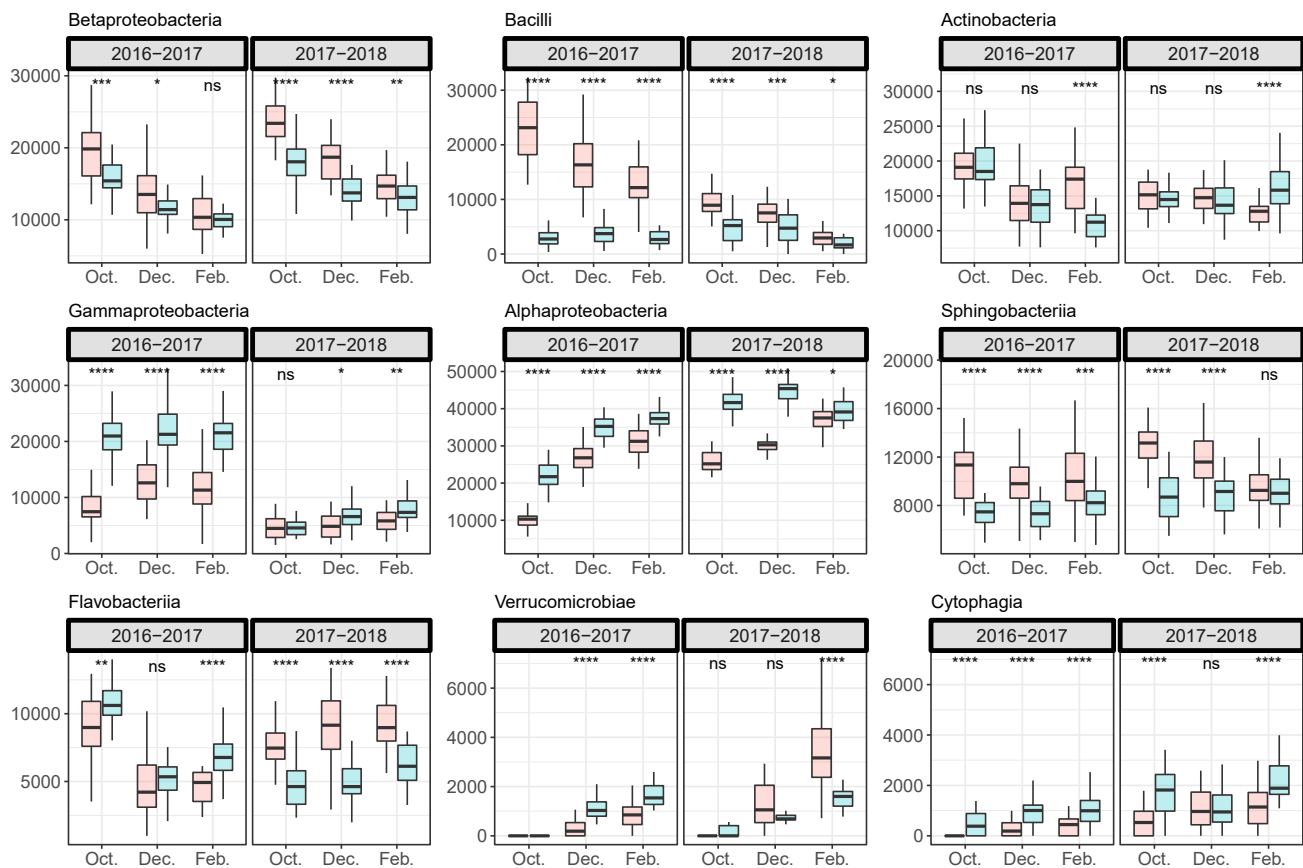

B

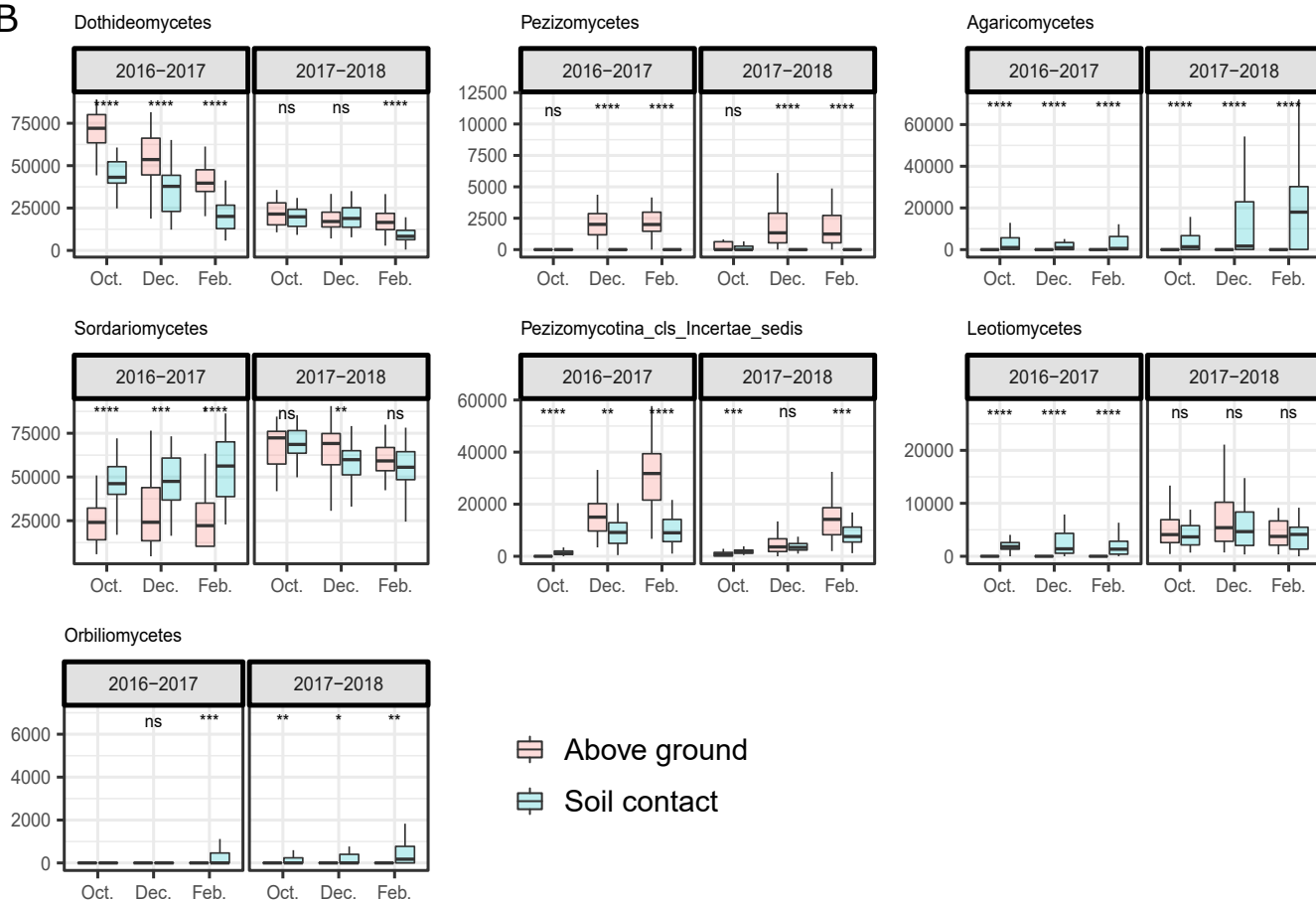

Supplement: Supplementary file 5 — Figure S2. Seasonal shift, from October to February, in the relative abundance of a selection of bacterial (A) and fungal (B) classes present on wheat residues (originating from wheat plants inoculated and not inoculated with Zymoseptoria tritici) according to cropping season (2016–2017, 2017–2018) and soil contact condition (in contact with the soil or above ground). Each box represents the distribution of class relative abundances for the 15 sampling points per treatment. Wilcoxon tests were performed for soil contact condition (NS: not significant; * p-value < 0.05; ** p-value < 0.01; *** p-value < 0.001). (PDF 60 kb) [file 40168_2019_736_MOESM5_ESM.pdf]

**A**

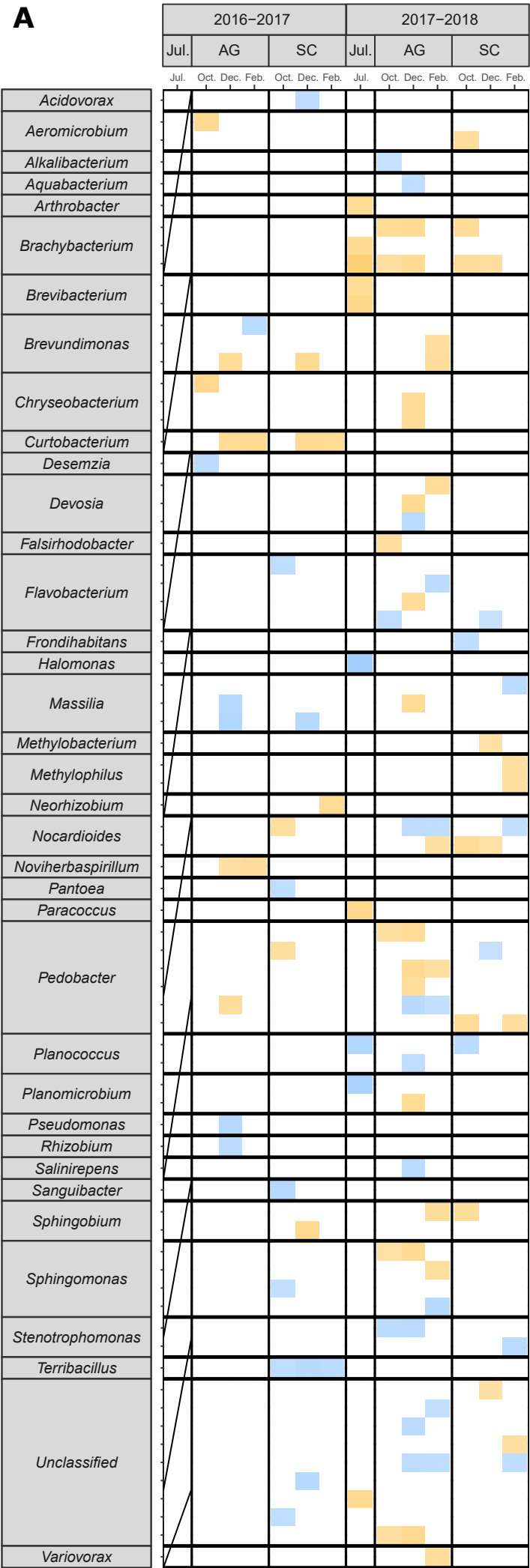

**B**

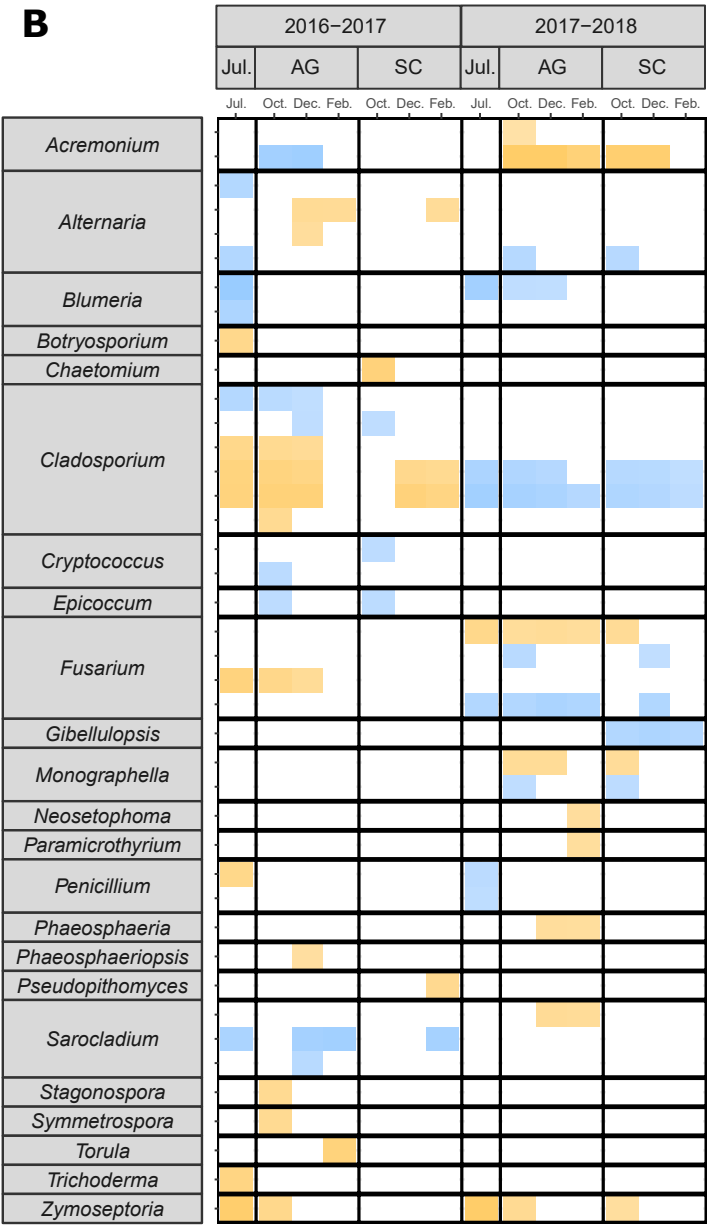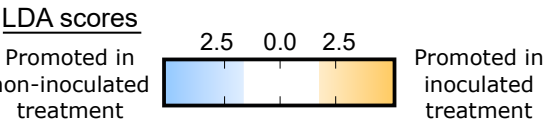

Supplement: Supplementary file 6 — Figure S3 Significant differences in the dominance of fungal and bacterial genera between wheat residues originating from inoculated (orange) and non-inoculated (blue) wheat plants in linear discriminant analyses (LDA), according to three sets of experimental conditions (cropping season, soil contact, seasonality). Only ASVs with p-values < 0.01 for the Kruskal-Wallis test and LDA scores > 2 are displayed. (PDF 263 kb) [file 40168_2019_736_MOESM6_ESM.pdf]

2016-2017

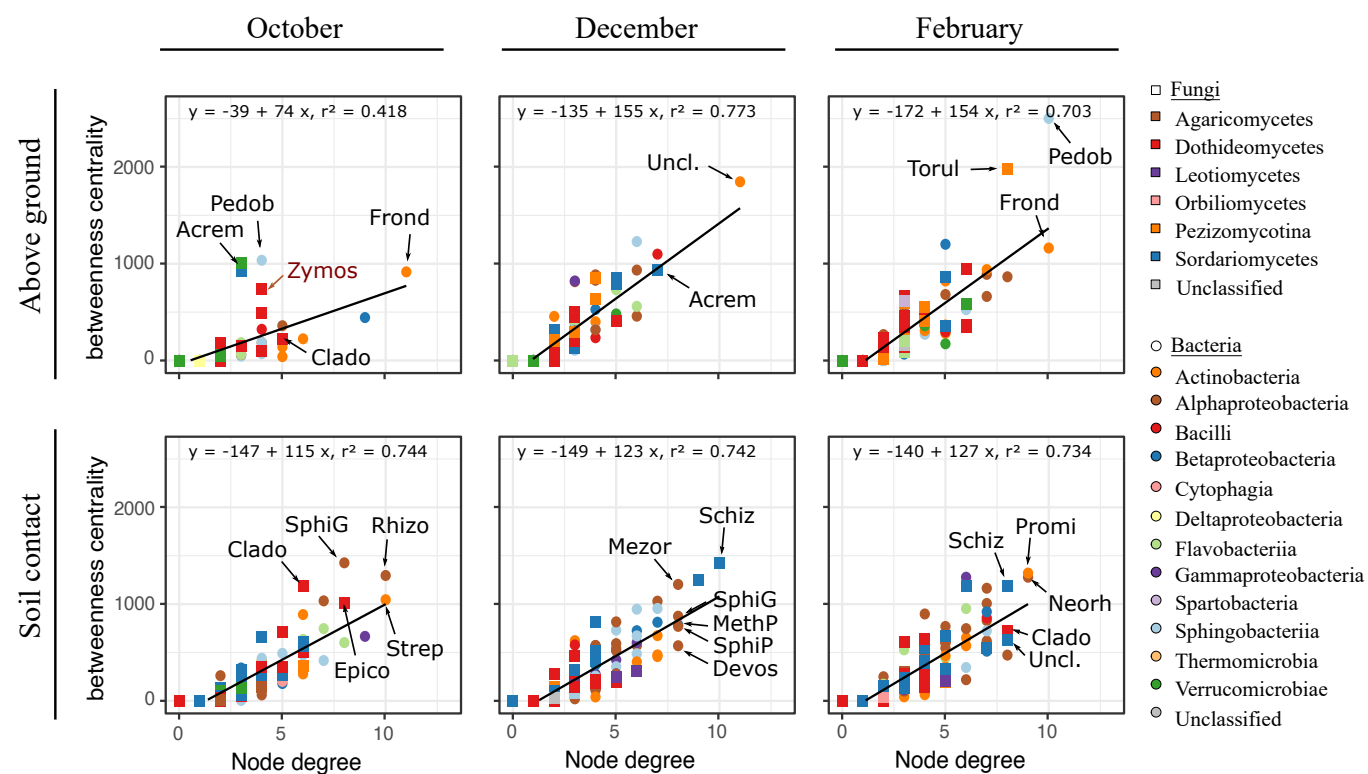

2017-2018

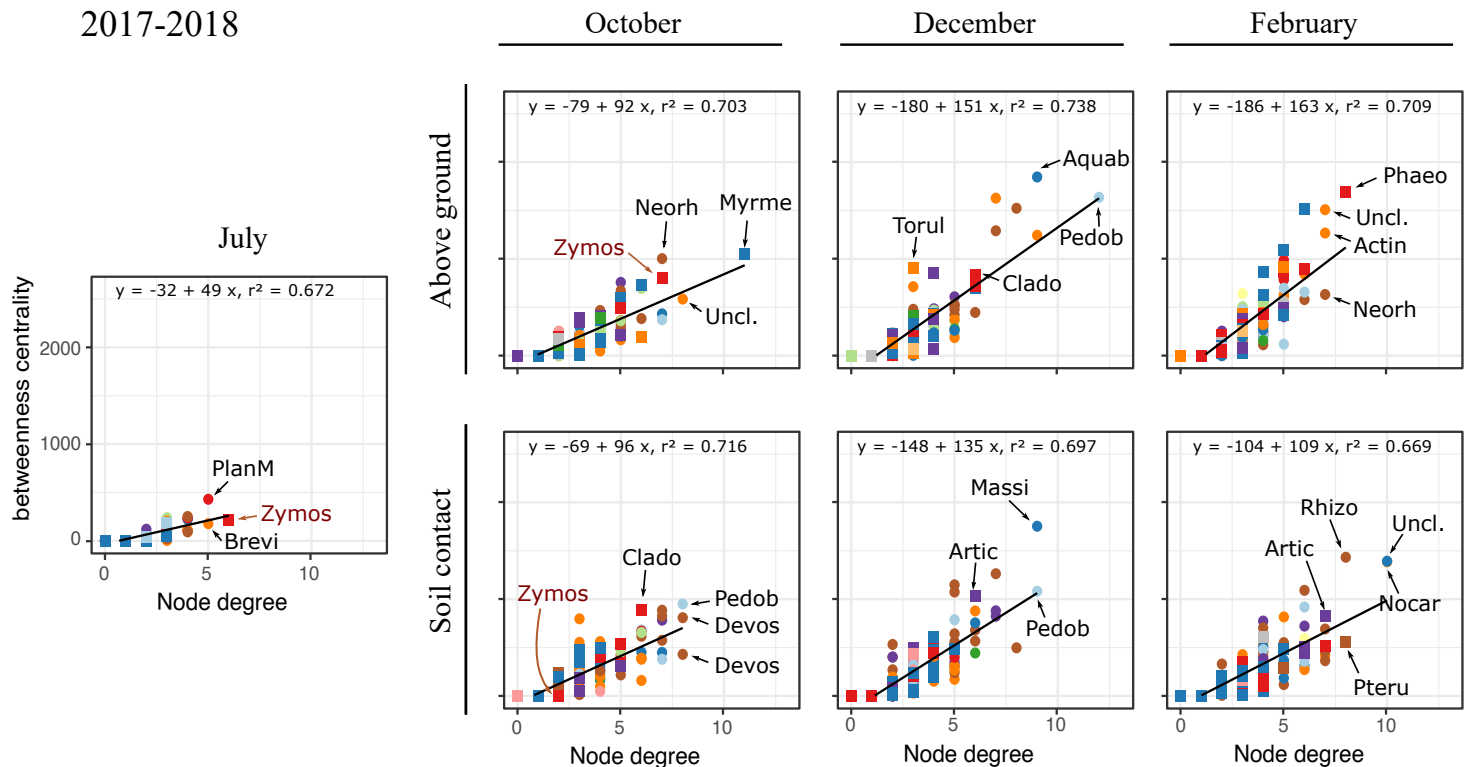

Supplement: Supplementary file 8 — Figure S5. Betweenness, centrality and degree of each ASV in the networks. Nodes with high betweenness, centrality and high degree values are considered to be keystone taxa in the networks. The genera of the fungal and bacterial ASVs with the highest degree and centrality are indicated: Acrem(onium); Actin(oplanes); Aquab(acterium); Artic(ulospora); Brevi(bacterium); Clado(sporium); Devos(ia); Epico(ccum); Frond(ihabitans); Massi(lia); Mesor(hizobium); MethP(=Methylophilus); Myrme(cridium); Neorh(izobium); Nocar(dioides); Pedob(acter); Phaeo(sphaeria); PlanM(=Planomicrobium); Promi(cromonospora); Pteru(la); Rhizo(bium); Schiz(othecium); SphiG(=Sphingomonas); SphiP(=Sphingopyxis); Strep(tomyces); Torul(a); Uncl.(assified); Zymos(eptoria). (PDF 122 kb) [file 40168_2019_736_MOESM8_ESM.pdf]
