# Supplementary material for: Differential dynamics of microbial community networks help identify microorganisms interacting with residue-borne pathogens: the case of Zymoseptoria tritici in wheat
Source: Microbiome. 2019 Aug 30;7:125. doi: 10.1186/s40168-019-0736-0 (PMC6717385; doi:10.1186/s40168-019-0736-0)
Supplement: Supplementary file 7 — Figure S4. Interaction networks based on bacterial and fungal ASVs combined for July (no contact with soil) for each cropping season (2016-2017, 2017-2018). Circles and squares correspond to bacterial and fungal ASVs, respectively, with colors represent classes. Isolated nodes are not shown. Edges represent positive (green) or negative (red) interactions. (PDF 38 kb) [file 40168_2019_736_MOESM7_ESM.pdf]

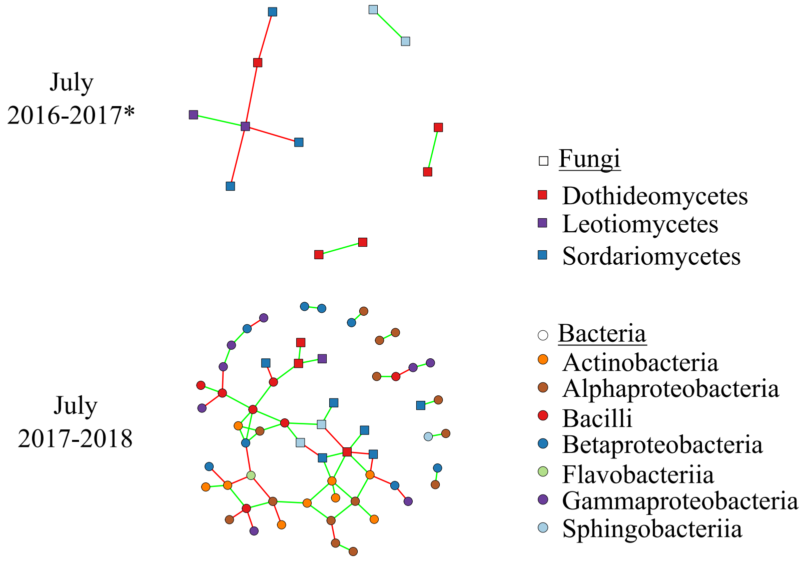

* only fungal ASV
