# Supplementary material for: Differential dynamics of microbial community networks help identify microorganisms interacting with residue-borne pathogens: the case of Zymoseptoria tritici in wheat
Source: Microbiome. 2019 Aug 30;7:125. doi: 10.1186/s40168-019-0736-0 (PMC6717385; doi:10.1186/s40168-019-0736-0)
Supplement: Supplementary file 9 — Figure S6. Mocks analysis for the two fungal sequencing runs (A, B, C) and the two bacterial sequencing runs (D, E, F). (A, D) Composition of the mocks. All microbial DNAs were pooled at equimolar concentrations. (B, E) Filter on the relative abundance of ASVs. The library size was normalized by proportion before analysis. The red line corresponds to a threshold at 3 ‰ of the size of the library. (C, F) ASVs detected in each mock. The 40 most abundant fungal ASVs are indicated (C), whereas all bacterial ASVs are indicated (F). The name of the ASVs corresponds to the taxonomic affiliation to the genus. All genera present in fungal mocks were detected (Gibberella and Fusarium are synonymous), while some bacterial genera were not detected in bacterial mocks, which differed only from one ASV. The red line corresponds to a threshold at 3 ‰ of the size of the library. (PDF 123 kb) [file 40168_2019_736_MOESM9_ESM.pdf]

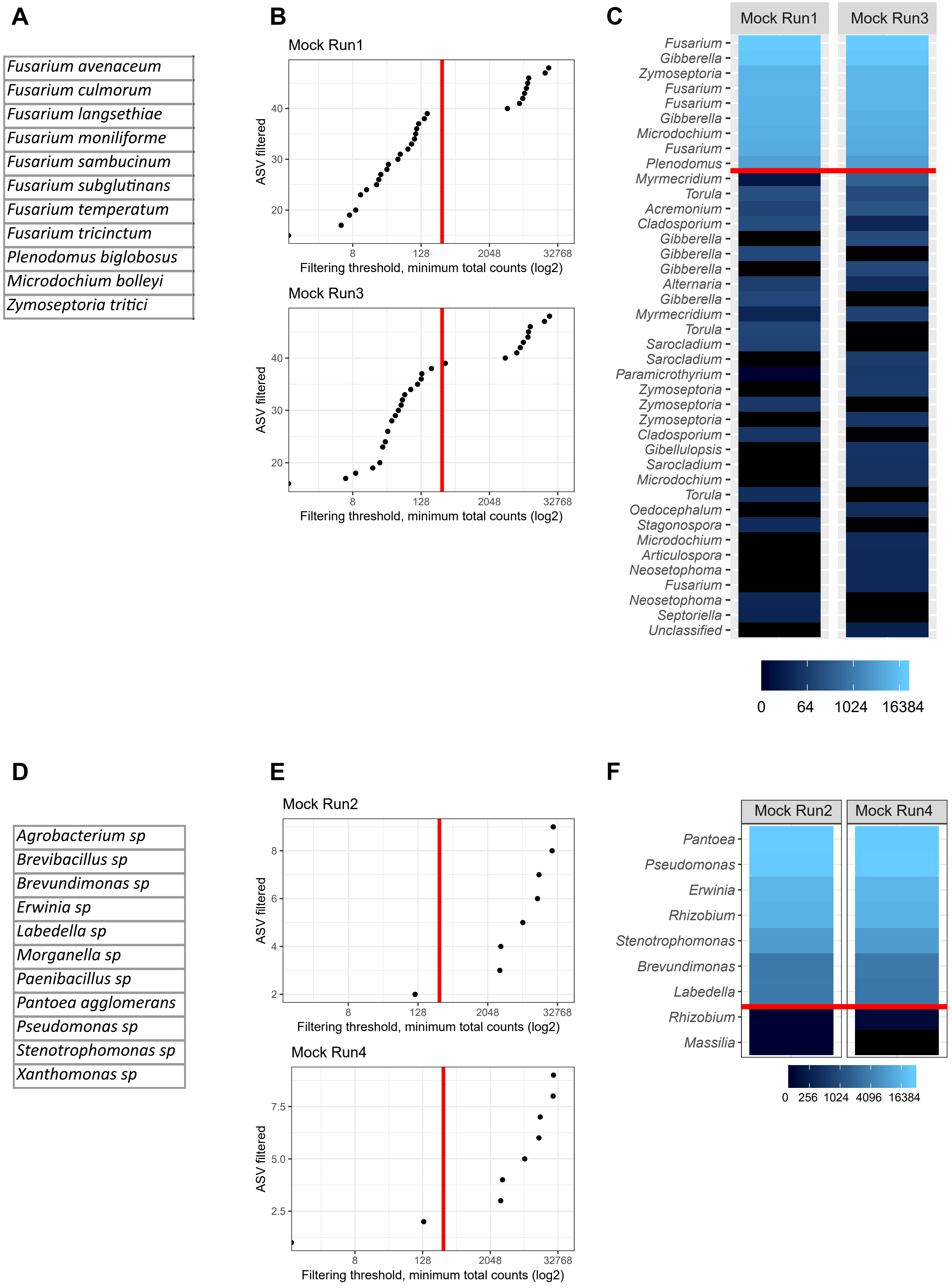
A
Fusarium avenaceum
Fusarium culmorum
Fusarium langsethiae
Fusarium moniliforme
Fusarium sambucinum
Fusarium subglutinans
Fusarium temperatum
Fusarium tricinctum
Plenodomus biglobosus
Microdochium bolleyi
Zymoseptoria tritici
B
Mock Run1
ASV filtered
40
30
20
8
128
2048
32768
Filtering threshold, minimum total counts (log2)
Mock Run3
ASV filtered
40
30
20
8
128
2048
32768
Filtering threshold, minimum total counts (log2)
C
Mock Run1
Mock Run3
Fusarium
Gibberella
Zymoseptoria
Fusarium
Fusarium
Gibberella
Microdochium
Fusarium
Plenodomus
Myrmecridium
Torula
Acremonium
Cladosporium
Gibberella
Gibberella
Gibberella
Alternaria
Gibberella
Myrmecridium
Torula
Sarocladium
Sarocladium
Paramicrothyrium
Zymoseptoria
Zymoseptoria
Zymoseptoria
Cladosporium
Gibellulopsis
Sarocladium
Microdochium
Torula
Oedocephalum
Stagonospora
Microdochium
Articulospora
Neosetophoma
Fusarium
Neosetophoma
Septoriella
Unclassified
0
64
1024
16384
D
Agrobacterium sp
Brevibacillus sp
Brevundimonas sp
Erwinia sp
Labedella sp
Morganella sp
Paenibacillus sp
Pantoea agglomerans
Pseudomonas sp
Stenotrophomonas sp
Xanthomonas sp
E
Mock Run2
ASV filtered
8
6
4
2
8
128
2048
32768
Filtering threshold, minimum total counts (log2)
Mock Run4
ASV filtered
7.5
5.0
2.5
8
128
2048
32768
Filtering threshold, minimum total counts (log2)
F
Mock Run2
Mock Run4
Pantoea
Pseudomonas
Erwinia
Rhizobium
Stenotrophomonas
Brevundimonas
Labedella
Rhizobium
Massilia
0
256
1024
4096
16384
